# The Monarch Initiative in 2024: an analytic platform integrating phenotypes, genes and diseases across species

**DOI:** 10.1093/nar/gkad1082

**Published:** 2023-11-24

**Authors:** Tim E Putman, Kevin Schaper, Nicolas Matentzoglu, Vincent P Rubinetti, Faisal S Alquaddoomi, Corey Cox, J Harry Caufield, Glass Elsarboukh, Sarah Gehrke, Harshad Hegde, Justin T Reese, Ian Braun, Richard M Bruskiewich, Luca Cappelletti, Seth Carbon, Anita R Caron, Lauren E Chan, Christopher G Chute, Katherina G Cortes, Vinícius De Souza, Tommaso Fontana, Nomi L Harris, Emily L Hartley, Eric Hurwitz, Julius O B Jacobsen, Madan Krishnamurthy, Bryan J Laraway, James A McLaughlin, Julie A McMurry, Sierra A T Moxon, Kathleen R Mullen, Shawn T O’Neil, Kent A Shefchek, Ray Stefancsik, Sabrina Toro, Nicole A Vasilevsky, Ramona L Walls, Patricia L Whetzel, David Osumi-Sutherland, Damian Smedley, Peter N Robinson, Christopher J Mungall, Melissa A Haendel, Monica C Munoz-Torres

**Affiliations:** Department of Biomedical Informatics, University of Colorado Anschutz Medical Campus, Aurora, CO 80045, USA; Department of Biomedical Informatics, University of Colorado Anschutz Medical Campus, Aurora, CO 80045, USA; Independent Consultant, Semanticly, Athens, Greece; Department of Biomedical Informatics, University of Colorado Anschutz Medical Campus, Aurora, CO 80045, USA; Department of Biomedical Informatics, University of Colorado Anschutz Medical Campus, Aurora, CO 80045, USA; Department of Biomedical Informatics, University of Colorado Anschutz Medical Campus, Aurora, CO 80045, USA; Environmental Genomics and Systems Biology, Lawrence Berkeley National Laboratory, Berkeley, CA 94720, USA; Department of Biomedical Informatics, University of Colorado Anschutz Medical Campus, Aurora, CO 80045, USA; Department of Biomedical Informatics, University of Colorado Anschutz Medical Campus, Aurora, CO 80045, USA; Environmental Genomics and Systems Biology, Lawrence Berkeley National Laboratory, Berkeley, CA 94720, USA; Environmental Genomics and Systems Biology, Lawrence Berkeley National Laboratory, Berkeley, CA 94720, USA; Data Collaboration Center, Critical Path Institute, Tucson, AZ 85718, USA; STAR Informatics, Delphinai Corporation, Sooke, BC V9Z 0M3, Canada; Biology, University of Fribourg, Fribourg, Switzerland; Environmental Genomics and Systems Biology, Lawrence Berkeley National Laboratory, Berkeley, CA 94720, USA; European Bioinformatics Institute (EMBL-EBI), Hinxton CB10 1SD, UK; College of Public Health and Human Sciences, Oregon State University, Corvallis, OR 97331, USA; Schools of Medicine, Public Health, and Nursing, Johns Hopkins University, Baltimore, MD 21205, USA; Department of Biomedical Informatics, University of Colorado Anschutz Medical Campus, Aurora, CO 80045, USA; European Bioinformatics Institute (EMBL-EBI), Hinxton CB10 1SD, UK; Dipartimento di Informatica, Università degli Studi di Milano Statale, Milano, Italy; Environmental Genomics and Systems Biology, Lawrence Berkeley National Laboratory, Berkeley, CA 94720, USA; Data Collaboration Center, Critical Path Institute, Tucson, AZ 85718, USA; Department of Biomedical Informatics, University of Colorado Anschutz Medical Campus, Aurora, CO 80045, USA; William Harvey Research Institute, Queen Mary University of London, London EC1M 6BQ, UK; Department of Biomedical Informatics, University of Colorado Anschutz Medical Campus, Aurora, CO 80045, USA; Department of Biomedical Informatics, University of Colorado Anschutz Medical Campus, Aurora, CO 80045, USA; European Bioinformatics Institute (EMBL-EBI), Hinxton CB10 1SD, UK; Department of Biomedical Informatics, University of Colorado Anschutz Medical Campus, Aurora, CO 80045, USA; Environmental Genomics and Systems Biology, Lawrence Berkeley National Laboratory, Berkeley, CA 94720, USA; Department of Biomedical Informatics, University of Colorado Anschutz Medical Campus, Aurora, CO 80045, USA; Department of Biomedical Informatics, University of Colorado Anschutz Medical Campus, Aurora, CO 80045, USA; Department of Biomedical Informatics, University of Colorado Anschutz Medical Campus, Aurora, CO 80045, USA; European Bioinformatics Institute (EMBL-EBI), Hinxton CB10 1SD, UK; Department of Biomedical Informatics, University of Colorado Anschutz Medical Campus, Aurora, CO 80045, USA; Data Collaboration Center, Critical Path Institute, Tucson, AZ 85718, USA; Data Collaboration Center, Critical Path Institute, Tucson, AZ 85718, USA; Department of Biomedical Informatics, University of Colorado Anschutz Medical Campus, Aurora, CO 80045, USA; European Bioinformatics Institute (EMBL-EBI), Hinxton CB10 1SD, UK; William Harvey Research Institute, Queen Mary University of London, London EC1M 6BQ, UK; The Jackson Laboratory for Genomic Medicine, Farmington, CT 6032, USA; Environmental Genomics and Systems Biology, Lawrence Berkeley National Laboratory, Berkeley, CA 94720, USA; Department of Biomedical Informatics, University of Colorado Anschutz Medical Campus, Aurora, CO 80045, USA; Department of Biomedical Informatics, University of Colorado Anschutz Medical Campus, Aurora, CO 80045, USA

## Abstract

Bridging the gap between genetic variations, environmental determinants, and phenotypic outcomes is critical for supporting clinical diagnosis and understanding mechanisms of diseases. It requires integrating open data at a global scale. The Monarch Initiative advances these goals by developing open ontologies, semantic data models, and knowledge graphs for translational research. The Monarch App is an integrated platform combining data about genes, phenotypes, and diseases across species. Monarch's APIs enable access to carefully curated datasets and advanced analysis tools that support the understanding and diagnosis of disease for diverse applications such as variant prioritization, deep phenotyping, and patient profile-matching. We have migrated our system into a scalable, cloud-based infrastructure; simplified Monarch's data ingestion and knowledge graph integration systems; enhanced data mapping and integration standards; and developed a new user interface with novel search and graph navigation features. Furthermore, we advanced Monarch's analytic tools by developing a customized plugin for OpenAI’s ChatGPT to increase the reliability of its responses about phenotypic data, allowing us to interrogate the knowledge in the Monarch graph using state-of-the-art Large Language Models. The resources of the Monarch Initiative can be found at monarchinitiative.org and its corresponding code repository at github.com/monarch-initiative/monarch-app.

## Graphical Abstract

**Graphical Abstract:**
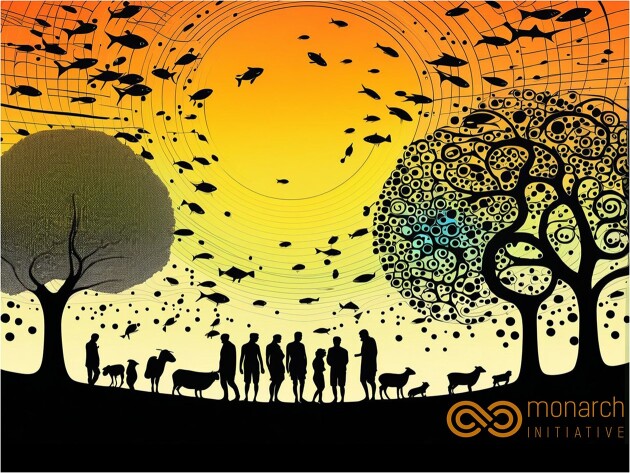

## Introduction

The Monarch Initiative is an international consortium that aims to harmonize data across scientific disciplines to reveal disease mechanisms and aid disease diagnosis. Fundamentally, there exist a wide variety of basic and translational data sources that are currently disconnected, making disease discovery challenging. Using innovative semantic engineering and data harmonization techniques and developing globally renonwned standards, the Monarch Initiative can help reveal unforseen mechanisms and fill gaps in our collective disease knowledge. The scope of Monarch is phenomic knowledge in all its forms, including human and model organisms phenotypic data, intending to elucidate the causes and mechanisms of human disease. Monarch's unique contribution to this phenomic-driven discovery is the development of novel methods for applying such analysis across a broad range of species to improve human health.

Updates and innovations since our last submission to the Database Issue of NAR in 2019 (1) include a recently modernized and simplified knowledge graph (KG) that integrates gene, phenotype, and disease data and a completely renovated user interface (UI) that has been redesigned based on extensive user research. Together, these components form the new Monarch Application. Our new design considers better accessibility for all users. It highlights a rebranded phenotype profile search tool—the Phenotype Explorer—and centrally integrates it into the application. We now provide site navigation highlighting Monarch's linked-data nature through breadcrumbs that show users the path they have taken while exploring the data captured in the Monarch KG.

To maximize data and resource availability, we make all Monarch resources freely available to everyone, minimizing barriers to entry. We don’t require anyone to register to use our website, and we provide multiple avenues for access to and reuse of Monarch's harmonized data, including APIs and bulk downloads. All of Monarch's code and standards are shared openly on GitHub, and the entirety of the Monarch platform is reproducible by others.

The Monarch KG, website, and the suite of tools described below fill a critical gap in the availability of gene-to-phenotype data by enabling *integration*—maximizing data reuse from a disparate landscape of disease and phenotypic knowledge and data sources, and supporting *diagnostics*—helping to improve the lives of patients with rare diseases by facilitating diagnosis and identification of treatments.

## The Monarch application

Like its predecessors, we developed the new Monarch Application (App) to enable mechanism discovery across species, revealing an understanding of the phenotypic diversity of life. It supports cross-species gene-to-phenotype data and semantic services for search, classification, and data harmonization. The Monarch App is backed by the Monarch Knowledge Graph (KG), which comprises the combined knowledge of 33 biomedical resources and biomedical ontologies (see *Data Sources* below), and is updated with the latest data from each source once a month. In the following sections, we describe the new architecture of the Monarch App and updates to the user interface before describing the latest updates to the Monarch KG and the KG Development process.

### Core components

The Monarch App includes a web-based user interface (UI) to browse Monarch's modernized KG and a RESTful Application Programming Interface (API) to retrieve data from this graph. The Monarch web application lets users explore the Monarch data and run analyses (e.g. phenotype profile search) through a graphical interface. We also publicly serve Monarch data through our API and file server. The API provides indexed data via a Solr instance and on-demand procedures through the Python implementation layer, allowing advanced graph querying and lookup capabilities. For users who want to integrate Monarch's KG into their own datasets or analysis pipelines, we use Knowledge Graph Exchange (KGX(2)) tools to serialize to various formats (e.g. SQLite, Neo4J, RDF, KGX). In this reimagined version of the Monarch infrastructure, we have put emphasis on transparency and reusability, leveraging the KGX format as a more user-friendly source of truth than the RDF outputs we previously used.

**Figure 1.**
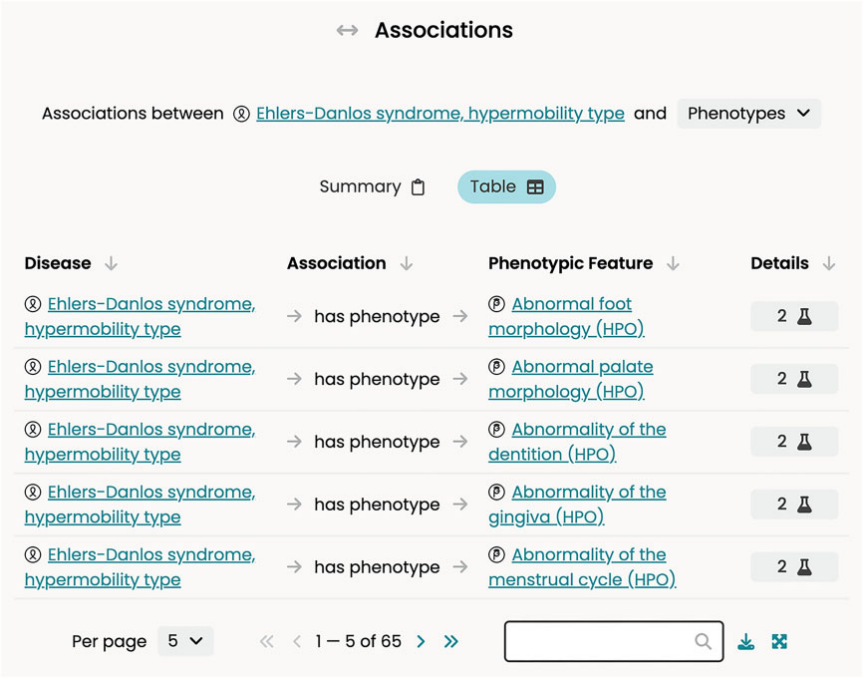
Example User Interface page. This interactive table of Ehlers-Danlos syndrome (EDS) Phenotypes (the currently selected association type) includes association types, supporting evidence, and provenance in the Details section (which gets expanded below the association table) and Taxon of the association object when appropriate (e.g. Genes), with summary, table views, and breadcrumb navigation.

*User Interface*. Our website redesign focused on achieving an intuitive User Experience that followed established design paradigms to align with user expectations (Figure 1 shows an example interactive table for phenotype associations of Ehler-Danlos syndrome (EDS)). We conducted over 20 hours of interviews with Monarch users to determine what worked well in the previous website version and what could be improved. A set of questions allowed us to evaluate users’ challenges, their level of confusion, and the use of features that were heavily relied upon or expected. We recorded the interviews on video and notes that we later distilled into a design document, based on which we created a comprehensive, annotated design mockup using the Figma platform (figma.com). Notable design changes and additions are listed in Table 1.

**Table 1.** Design highlights of the new Monarch Initiative website

Design highlights of the new Monarch Initiative website
The front page features videos advertising the website's core features and illustrating how to use them.
Tabular data were condensed into summary views to avoid overwhelming the user with too much information at once while allowing them to explore the data further using alternate views.
Page layout has been simplified to organize information more clearly and make workflows for using features more obvious.
Breadcrumbs highlight the linked nature of the data by showing the path users take through the KG while browsing nodes.
Redesigned auxiliary pages, e.g. *‘About’* and *‘Help,’* now better catalog the range of Monarch's resources.
Recognizing its unique value, we reworked and rebranded the *‘Phenotype Profile Search’* tool, now the *‘Phenotype Explorer,’* more prominently featured on the homepage. We reimagined the previous sequence of ‘wizard’ prompts for easier use as a concurrently visible and more standard search layout.

The website is a single-page application written in Vue 3 (vuejs.org), using the composition API for modularity, flexibility, and simplicity. TypeScript (typescriptlang.org) is used for type safety, an essential feature given the code base's size and the data structures' complexity. ESLint (eslint.org) and Prettier (prettier.io) enforce code consistency, readability, and use of recommended patterns. To ensure the quality and stability of the website over short- and long-term maintenance cycles, we’ve written a suite of automatic tests that check types, linting, formatting, accessibility, component functionality (unit), and high-level functionality (end-to-end). The industry standard tool Axe by Deque (deque.com/axe) is used to check the website for common accessibility problems automatically.

Monarch's new UI follows important tenets of web design. *Responsive design* ensures that the website looks and behaves on mobile devices as well as it does on desktop devices. *Accessible design* ensures that users with sight/motor/etc. disabilities can navigate and operate the website without difficulty. A *feedback form* accessible from the corner of the screen at all times allows users to submit comments and questions to our helpdesk, with troubleshooting details automatically included. A separate *beta version of the website* is deployed alongside the production version, allowing users to test new features before we finalize them. These additions allow us to iterate and adapt to users' needs quickly.

*Monarch API*. The Monarch Application Programming Interface (API) is a RESTful API that wraps functionality required by the Monarch App, including the retrieval of disease, gene, and phenotype associations, phenotypic profile comparisons, and other graph operations. The previous Monarch API specification design provided explicit endpoints for every combination of entities in the Monarch Graph; the new Monarch API provides a concise set of abstracted endpoints that allow the same functionality with reduced complexity (Table 2).

**Table 2.** Endpoints of the Monarch API

Endpoint	Label	Summary and Examples
/v3/api/entity/{id}	*Get Entity*	Retrieves summary info about an entity (node). E.g., the identifier ‘entity/*HGNC:992*″ retrieves details about the human gene ‘*BCL2 like 1*.’
/v3/api/entity/{id}/{category}	*Association Table*	Retrieves associations of a given entity by category. E.g., ‘*entity/HGNC:992/phenotypes*’ returns all phenotypes associated with ‘*BCL2 like 1*.’
/v3/api/association	*Get Associations*	Retrieves all associations for an entity or between two entities. E.g., ‘*/association/predicate = biolink:has_phenotype&object = HP:0 000 003″* returns all entities with the phenotype ‘*Multicystic kidney dysplasia.’*
/v3/api/search	*Search*	Searches for entities by label, with optional filters.
/v3/api/autocomplete	*Autocomplete*	Autocompletes for entities by label.
/v3/api/histopheno/{id}	*Histopheno*	Retrieves the high-level ancestor classes and counts for a given phenotype (primarily used in the Monarch UI).
/v3/api/semsim/compare/{subjects}/{objects}	*Compare*	Assesses pairwise similarity and returns a score for two sets of phenotypes. E.g., the collection of phenotypes known for the diseases ‘*Ehlers-Danlos, hypermobility type*’ and ‘*aneurysm-osteoarthritis syndrome*’ are 85% similar, sharing phenotypes such as *Osteoarthritis*, *Mitral valve prolapse*, *Striae distensae*, *Scarring*, *Joint dislocation*, *Soft skin*, and *Joint laxity*.

### The Monarch knowledge graph

A KG, or semantic network, represents a network of entities—e.g. events, situations, or concepts– and illustrates the relationship between them. The Monarch KG comprises two major components: a set of data source ingests and ontologies. The components of the Monarch KG and the associations between them are represented as nodes, edges, and labels. A *data source ingest* imports data from resources such as the external Panther Database (3) or Monarch's disease annotations to Human Phenotype Ontology terms (HPOA (4)) and transforms them into the Monarch KG schema. As described below, ontologies are integrated into a ‘semantic layer,’ a Biolink-conformant representation of the Phenomics Integrated Ontology (PHENIO; github.com/monarch-initiative/phenio), which serves as a hierarchical schema and classification system for the integrated data.

The new Monarch KG integrates gene, disease, and phenotype data. The previous version of the graph included additional data types, such as gene variants and genotypes, and their associations; however, the resulting complexity sometimes made it difficult to derive meaningful knowledge from the graph. These data are still available but do not form the primary KG classification backbone.

*The KG Data Model*. We have adopted Biolink (5) as our KG’s data model. The Biolink Model provides a standardized representation of biological entities (genes, disease, phenotype, etc.) and their associations. We developed Biolink in the context of the NCATS Biomedical Data Translator Project (6)—which aims to integrate the vast amounts of currently available biological, biochemical, and biomedical research data and accelerate the treatment of disease.

**Figure 2.**
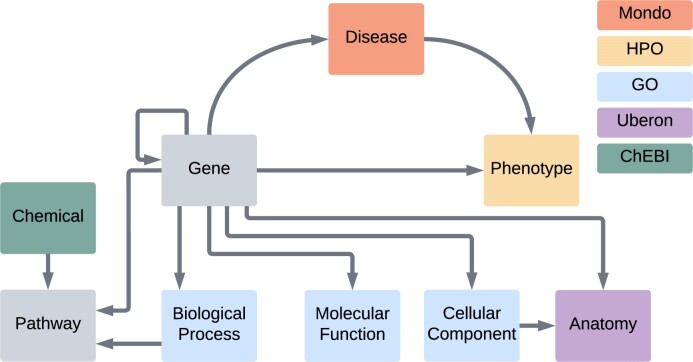
Overview of the Monarch data model. The Monarch Subset of the Biolink Data Model centered around Gene, Disease and Phenotype Associations. Colors indicate which ontology the category of node comes from. Gene and Pathway are ingested data types, not ontology concepts.

The Monarch KG implements a subset of the Biolink Model with a core set of gene, disease, and phenotype associations. In addition, we include associations to Gene Ontology (GO), Anatomy, Chemical and Pathway terms (Figure 2).

**Figure 3.**
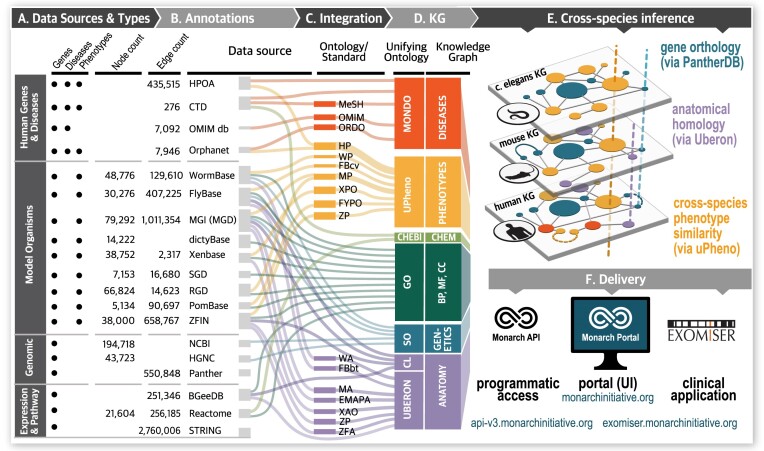
Data harmonization within the Monarch KG. The three primary data types in the Monarch KG are genes, diseases and phenotypes (**A**).This image details their entity (node) and link (edge) counts and the unifying ontologies (**D**) by which the source data (**B**) and ontologies (**C**) are harmonized. Cross-species inference (**E**) is accomplished via gene orthology, homology and phenotype similarity. Content dissemination (**F**) is via API, the Monarch UI and within the clinical application Exomiser. Note that the figure expresses only a portion of the integrated ontologies (column C). For a comprehensive list see PHENIO documentation (linked below). In Column D, GO: Gene Ontology; BP: Biological Process; MF: Molecular Function; CC: Cellular Component.

*Data Sources*. The Monarch KG integrates knowledge from 33 heterogeneous data sources, including research organism knowledgebases (e.g. MGD (7), ZFIN (8), WormBase (9), FlyBase (10), Xenbase (11) SGD (12), PomBase (13), DictyBase (14) and BGeeDB (15)), catalogs of human models of disease (Mondo (16)) and gene-to-disease information (OMIM(17), Orphanet (orpha.net/consor/cgi-bin/index.php), information about signaling and metabolic pathways (Reactome (18)), protein-to-protein association networks (STRING (19)), and other ontologies (HPO (4) with abnormal human phenotypes, Gene Ontology (20) with gene functional annotations, and PHENIO with integrated data about phenotypes across species and genetic backgrounds; also below) (Figure 3). We note that, historically, we utilized individual model organism database resources, however, since the Alliance of Genome Resources (30) has come into fruition, we now leverage their pre-harmonized genotype-phenotype data. A complete list of the sources ingested into the Monarch KG and how to cite them can be found at monarch-initiative.github.io/monarch-ingest/Sources.

*‘Semantic Layer’: The Phenomics Integrated Ontology (PHENIO)*. Ontologies provide a semantic, hierarchical schema for integrated data. For example, the KG incorporates a gene-to-phenotype association between the gene *ADGRV1* (HGNC:17416(21)) and the disease term ‘*Usher syndrome type 2C*’ (MONDO:0011558). Mondo(16) groups ‘*Usher syndrome type 2C*’ under ‘*Usher syndrome’* (MONDO:0019501), itself grouped under ‘*syndromic retinitis pigmentosa*’ (MONDO:0020240). These groupings enable us to find the gene *ADGRV1* when searching for all gene associations related to the Mondo terms ‘*Usher syndrome’* and ‘*syndromic retinitis pigmentosa*.’

We leverage the relational structure of ontologies to enable semantic similarity algorithms that power critical applications such as variant prioritization, phenotypic profile search, and more. We created the Phenomics Integrated Ontology (PHENIO), which describes diseases, phenotypes, anatomical and chemical entities, and their relationships. PHENIO is built using modern ontology life-cycle management (Ontology Development Kit (ODK (22))), including quality control and release management. PHENIO integrates ontologies such as the Unified Phenotype Ontology (uPheno) for phenotypic abnormalities (23), Ontology of Biological Attributes (OBA) for traits (24), the Mondo Disease Ontology for diseases (16), Uberon for anatomical entities (25) and the Evidence and Conclusion Ontology (26), which categorizes different types of evidence used in scientific research. PHENIO includes all materialized relationships between all parent and child classes using the relation-graph package (github.com/INCATools/relation-graph), and PHENIO classes are annotated with categories from the Biolink Model. To seamlessly ingest PHENIO into the Monarch KG, it is translated into a KG form (i.e. nodes and edges following a graph model and aligned to types defined by Biolink), referred to as KG-Phenio (github.com/Knowledge-Graph-Hub/kg-phenio).

### Knowledge graph ingest system

**Figure 4.**
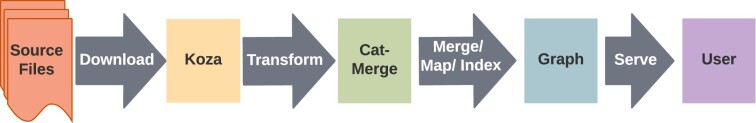
Monarch KG Construction Workflow. Source files are downloaded, passed through Koza for transformation to Biolink and KGX format, Cat-Merge for merging and node normalization, and finally served to the user through various access points.

**Figure 5.**
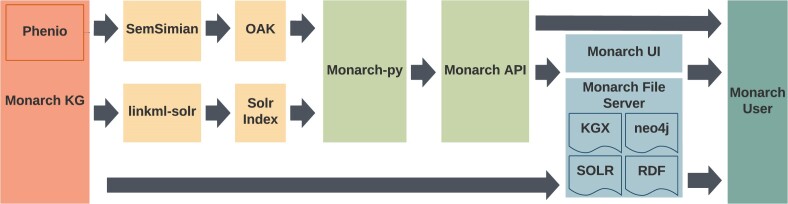
Expanded view of deploying the Monarch KG to the end user through the Monarch Python package, API, file server and web interface.

The Monarch KG is constructed in phases, from data download to transformation, merging, indexing, and data service (Figure 4); these are described below. The Monarch App depends on two representations of the KG: the Solr index, which drives the website's search function and page content, and an SQLite representation, which is exploited by the Ontology Access Kit (OAK(27)) to drive its semantic similarity-based functionality (phenotypic profile search, etc). Figure 5 shows the phases of the KG construction and their final representations.

Our ingest adheres to the following modeling principles: (i) conformance with the Monarch Biolink specification; (ii) normalization of nodes to ensure assignment of canonical prefixes to every node type; (iii) nodes should be ingested from their authoritative source, separate from edge ingests; (iv) equal emphasis on gene and protein representation, representing them as a single node or concept in the Monarch KG to emphasize a concise, manageable data model; (v) variant-to-disease or to-phenotype associations are rolled up to the gene level and (vi) gene-to-disease associations are well provenanced.

**Figure 6.**
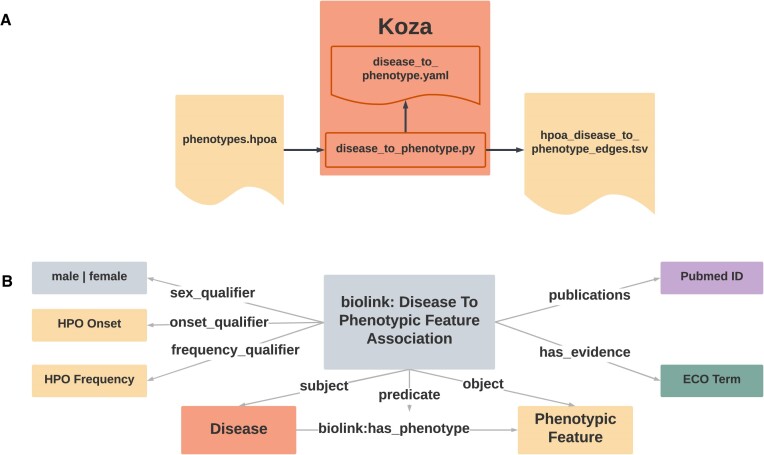
Extract-Load-Transform (ETL) with Koza. (**A**) Data flow in the Koza ingest for Human Phenotype Ontology disease-to-phenotype annotations (HPOA). Raw source files are transformed into KGX formatted tabular data. (**B**) The Biolink Model association for disease-to-phenotype relationships used in the HPOA Koza ingest.

*Download and Extract-Load-Transform (ETL)*. Data from each knowledge source are downloaded using a file caching process (github.com/monarch-initiative/kghub-downloader) to reduce repeated data transfer requests and increase reproducibility. To replace Monarch's previous ingest process (Dipper (28)), we developed Koza (koza.monarchinitiative.org), a Python-based ETL tool designed to support the ingest and curation of a heterogeneous landscape of data sources (Figure 6; which includes the data flow in the Koza ingest (A) and details of the example HPOA ingest (B)). Koza uses YAML configuration files to declare transformation and data model mapping specifications. This method minimizes source-specific logic and maintenance while improving readability and testability in the new pipeline.

*Entity mapping and merging*. Many sources use divergent identifier schemes to represent the same data types. For example, HPOA integrates disease information using OMIM (17), Orphanet, and Decipher (29) identifiers. In contrast, the Alliance of Genome Resources (30) refers to the same kinds of disease data using Disease Ontology (DO) (31) identifiers. KGs that underlie web apps for aggregating and analyzing information, such as the Monarch App, must ensure each concept (e.g. gene, disease, etc.) is represented by a single node in the graph. This requires entity merging; the example above requires merging disease concepts described by OMIM, Orphanet, or DO when they are the same. For example, cystic fibrosis has IDs *OMIM:219 700*, *Orphanet:586*, *NCIT:C2975*, *DOID:1485* and *ICD10CM:E84*.

We use the Simple Standard for Ontological Mappings (SSSOM(32)) to capture mappings in a standardized form. It provides a vocabulary to describe mapping provenance (author, sources, dates), evidence to substantiate the validity of a mapping, and a TSV-based representation for entity mappings. Mappings required for our KG integration are stored in the Monarch Mapping Commons (github.com/monarch-initiative/monarch-mapping-commons). Mappings between the gene identifiers are externally sourced and converted into SSSOM. We prioritize the naming source (e.g. HGNC) as our primary source for mappings to other identifier schemes (such as ENSEMBL (33)). If the naming source lacks a mapping for a specific identifier scheme, we defer to the source of the external identifier (the identifier scheme we map from). Disease mappings are provided by Mondo (16), which aims to integrate disease terminologies and ontologies such as DO, OMIM, ORDO, and NCIt (34) into a coherent computational framework of diseases. Mondo provides a computational framework that combines mutually inconsistent tnmappings from these external resources with algorithmically inferred and internally curated ones into a coherent and comprehensive ontology. In particular, mappings provided as imprecise cross-references are enhanced to make semantic precision explicit, e.g. exact, broad, or narrow match, where exact mappings can be used to populate the KG and perform analytics across resources. All mappings harmonized this way are integrated into the Monarch KG via the Monarch Mapping Commons.

Cat-merge (github.com/monarch-initiative/cat-merge) is a Python library that merges individual KGX node and association files from Monarch's ingest pipeline. It merges SSSOM mappings from the Monarch mapping commons into one node and one edge file in a tarball. Associations with subjects or objects that do not exist in our source-of-truth node set are set aside in a quality control data file that is analyzed to improve Monarch's node ingests and mapping sets.

*Quality reporting*. The quality assessment for the Monarch KG has been completely rewritten. Graph statistics are generated using the *Summarize Graph* tool from KGX. Additionally, we created the Monarch Quality Control (QC) Dashboard (monarch-initiative.github.io/monarch-qc) for quality metrics that are more specific to Monarch. QC reports are now generated separately by *Monarch QC Reports* instead of during the *Monarch KG Ingest Pipeline*, enabling retrospective analysis of older KG builds with new reporting features. We leverage LinkML code-generation tools to add new functionality to the QC report.

*KG Dissemination*. The data artifacts generated by the KG ingest pipeline (the KG and its individual components), the PHENIO database, and the Solr index are stored in versioned releases on a cloud bucket and made available at data.monarchinitiative.org. We use the KGX format (github.com/biolink/kgx) to share our Biolink-conformant KG. This makes it easy to combine the Monarch KG (or any of its parts) with other KGs from the KG-Hub Knowledge Graph Repository(35) or other projects that employ KGX and Biolink, such as the aforementioned NCATS Biomedical Translator.

## Analysis tools power by Monarch

### Clinical diagnostic resources

We developed *Semsimian* (github.com/monarch-initiative/semsimian), an OAK(27) plugin to measure the semantic similarity between sets of ontology terms; for example, the similarity between a patient's phenotypic features and those described for a particular disease. In addition to leveraging the classic, transparent measures of similarity such as Resnik (36) and Jaccard (37), Semsimian includes functionality to compare ontology term embeddings using cosine similarity. This enables us, for example, to leverage advanced methods for graph embeddings like those in the GRAPE package (38) to compare phenotypes using the Monarch KG. We have developed a standard format for disseminating semantic similarity profiles using LinkML (linkml.io) for downstream applications.

Our tools require regular data updates to fulfill the needs of Monarch's users. It is particularly critical for our flagship diagnostic tool, Exomiser (39), to have access to the latest disease-to-gene discoveries, phenotypic annotations, and ontologies as it is used in clinical diagnostic pipelines such as the Genomic Medicine Service of the UK’s National Health Service. Therefore, scalable approaches are required to generate and appropriately version semantic similarity profiles. Semsimian allows us to rapidly generate these profiles and experiment with different configurations of PHENIO (see *Core Components*) to identify the optimal way to support clinical diagnostics. We have implemented this comparative analysis using our Phenotypic Inference Evaluation Framework (PhEval; github.com/monarch-initiative/pheval), which evaluates the diagnostic yield for multiple variants of Exomiser with different semantic similarity profiles.

### Monarch ChatGPT plugin

Large Language Models (LLMs) are computational architectures representing massive collections of patterns found in human written communication. In recent years, particularly with the release of successively larger Generative Pre-training Transformer (GPT) models by OpenAI (openai.com), researchers and software engineers have found that these models can produce comprehensive and informative responses to various questions without requiring additional training (40). We are exploring the potential of LLMs to enable the use of plain English to ask questions and get answers from Monarch. However, LLMs must provide accurate output with few improvised (‘hallucinated’) details. One strategy is Retrieval Augmented Generation (RAG), whereby an LLM-based agent can search for information from an external source while responding to a user query (41). This strategy is used by Microsoft's new Bing (bing.com), which can complement its natural-language responses to user queries with collections of Internet sources and ChatGPT Plugins, which enable OpenAI’s ChatGPT to call external APIs.

**Figure 7.**
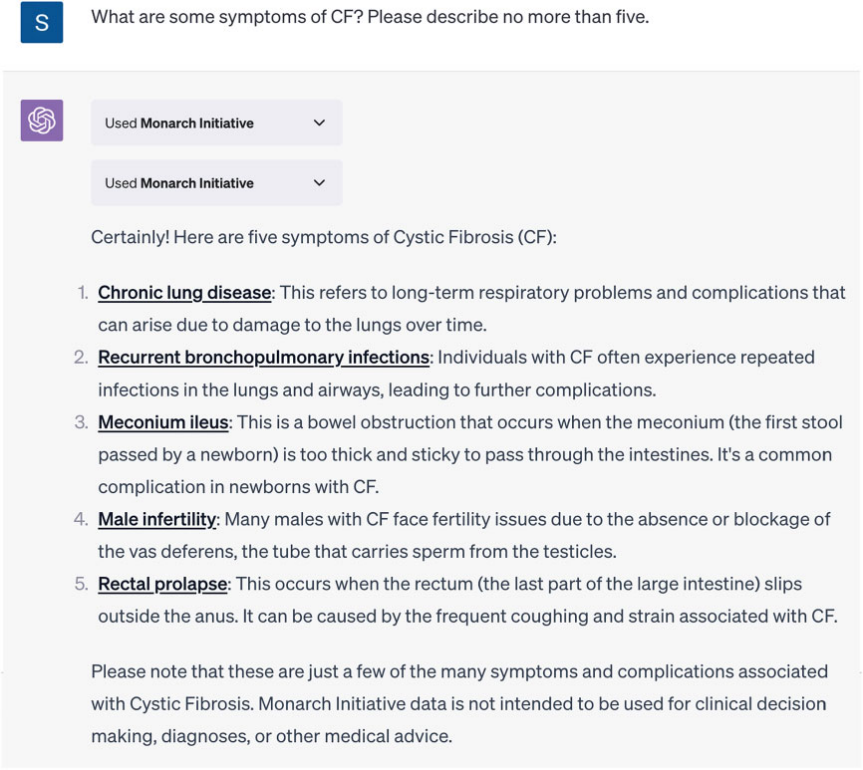
The Monarch ChatGPT plugin allows the Monarch KG to be queried and to return answers in natural language.

We developed an API for the Monarch KG suited for LLM-driven interactions that supports search and entity association retrieval based on the RAG pattern. The KG supplies the knowledge needed to respond to and contextualize user queries. Our new ChatGPT plugin allows ChatGPT subscribers to query the Monarch KG using natural language. This chat-based LLM interface supports complex questions previously thought intractable for search interfaces. For example, the query ‘*What symptoms are associated with CF*?’ (Figure 7) prompts the LLM to (i) search the Monarch KG for ‘*Cystic Fibrosis*’ to identify the corresponding Mondo identifier MONDO:0009061, (ii) retrieve a list of symptoms associated with that identifier and (iii) summarize the information for the user, providing links to Monarch resources for more information.

Alternatively, users may ask a more general question and refine it through the interface. Asking ‘*What diseases are associated with flaky skin?*’ will return no direct matches, but the interface will suggest related alternative symptoms (e.g. ‘*desquamation*’). While LLM-based systems show promise in academic research, supporting accuracy with external information such as that available within the Monarch KG will be crucial to realizing their full potential(42).

The plugin is available to subscribers of OpenAI’s ChatGPT Plus service, and can be found in the ChatGPT Plugin store by searching for ‘Monarch Initiative.’ A brief installation guide is available at the plugin repository, at github.com/monarch-initiative/oai-monarch-plugin. We are actively developing a non-subscription alternative, the Monarch Assistant, to improve access and feature integration. To complement our work to leverage LLM-based tools such as ChatGPT, we are also developing the OntoGPT (43) package (monarch-initiative.github.io/ontogpt) to leverage LLMs to extract data from unstructured biomedical text to offer new possibilities for AI-backed searching in Monarch.

### GRAPE integration: advanced graph machine learning

The Monarch KG is integrated into the GRAPE library(38) for graph analysis and machine learning (ML), allowing users to download any KG version, efficiently obtain metrics (e.g. connected components, the clustering coefficient, the closeness and harmonic centralities, and the graph diameter), and perform graph machine learning on the KG. Users can leverage several scalable graph ML models to run tasks such as node embedding (learning a meaningful vector representation for the nodes), node-label and edge-label prediction (predicting the class of the node or edge respectively), or link prediction (predicting whether a link between two nodes exists, for example to predict gene to disease relationships). The Monarch KG has been used in this way to investigate possible associations between diet and female reproductive disorders for further downstream analysis, and to compare the results of graph machine learning to non-graph machine learning techniques such as random forest(44).

## Community engagement

*Semantic Engineering Training*. The Monarch Initiative runs a fortnightly, free, and public Semantic Engineering seminar (oboacademy.github.io/obook/courses/monarch-obo-training). We cover subjects from ontology engineering and semantic mappings to using the Monarch KG. As part of our training, we document most of our tutorials as self-taught materials on OBO Academy(45), which is free, run by volunteers across the OBO Community, and supported by members of the Monarch team.

*Outreach*. We provide ongoing outreach to our community of biologists, bioinformaticists, and developers via multiple channels: publications, preprints, website, blog, social media (including a new LinkedIn page; linkedin.com/company/the-monarch-initiative), mailing lists, a YouTube channel, presentations at conferences, and workshops. Users can submit questions and bug reports via our GitHub issue tracker and Monarch Helpdesk. Developers can access our code and ontologies on GitHub and offer feedback. More details at monarchinitiative.org/outreach

*Mondo Outreach Series*. We host a monthly Mondo Outreach Series to disseminate disease-related information and available functionality of the Monarch application. This community event brings together stakeholders and clinical terminology experts, allowing Mondo users to discuss use cases and requirements. These discussions advise Mondo development and inform the Monarch team about disease integration required by the Monarch KG.

*Integration collaborations*. In addition to the partnerships we have cultivated with source data providers, we have worked with various large-scale projects and communities to integrate our KG and services in their projects for wider dissemination and impact of Monarch's harmonized data. These collaborations include co-founding the NCATS Biomedical Translator and National Covid Cohort Collaborative (N3C) and working closely with the Critical Path Institute, the Genetic and Rare Diseases Information Center (GARD), and the National Organization for Rare Disorders (NORD).

## Discussion

The Monarch Initiative is an international consortium that makes data interoperable through semantic technologies and ontology standards. A fundamental premise within Monarch is that we learn different things about the relationship between genotype and phenotype from different organisms. Therefore, we aim to collect, integrate, and make a broad compendium of species and sources computable. The lack of consistency in how different data sources curate associations, such as disease-to-gene or gene-to-phenotypes, is a significant challenge. Monarch has harmonized these data for computational reuse over a decade of great effort and community coordination. As a testament to the impact of Monarch's data reuse, the Monarch Initiative was awarded the NIH-FASEB Distinguished Achievement Award for Data Reuse in the first NIH DataWorks! Prize (herox.com/dataworks) in February of 2023. Core strategies include best practices for identifier management and reconciliation and community development of species-neutral ontologies such as Uberon for anatomy, Gene Ontology (GO) for function, and uPheno for phenotypes. Monarch has built some of the most widely used resources for disease knowledge standardization: Mondo, Phenopackets(46) (a GA4GH and ISO standard), and HPO. Phenotyping patients using HPO has increased diagnostic yield by over 20% using Monarch's semantically integrated data(47), which extends coverage of gene-to-phenotype associations in humans from ∼21% to ∼84%(28).

At its core, the Monarch App includes an ETL platform for ingesting, harmonizing, and serving diverse life science data relating genes, phenotypes, and diseases into a semantic KG for use in various downstream applications. We offer users data in various formats and applications, including APIs, file servers, Python packages, and a full-featured website for semantic navigation. Combining state-of-the-art technologies such as ontologies, knowledge graphs, and semantic similarity, we deliver tools that enable the retrieval and analysis of integrated knowledge from critical biomedical resources. We have built a flexible, robust, and resilient infrastructure, balancing our need to provide services at scale with minimal downtime, reducing cost, and improving sustainability.

Salient demonstrations of the need for the Monarch App and KG are their downstream use in diverse applications. We have greatly improved the utility of LLMs to retrieve data with high confidence by leveraging the Monarch KG to supply the knowledge needed to respond to and contextualize user queries, exemplified by our ChatGPT plugin. Monarch's Graph ML and semantic similarity-based analysis tools are also leveraged by various genetic diagnostic tools, such as Monarch's Exomiser variant prioritization tool, to improve the diagnostic yield. Monarch data reuse is notable for its scope and mechanism discovery across species and diverse domains such as rare disease diagnosis, evolutionary biology, veterinary medicine, and biodiversity. Monarch ontologies and resources are utilized by countless downstream open science and commercial applications, made possible by Monarch's extraordinary coordination and harmonization efforts to realize a truly integrated and computable suite of resources.

We also plan to expand Monarch's data ingests leveraging *Phenologs*: orthologous phenotypes between organisms as determined by overlapping sets of orthologous genes associated with each phenotype(48). We have created a data pipeline for calculating Phenologs using the Monarch KG; the ‘significant’ Phenologs (Phenologs with a p-value less than a calculated false-discovery rate) will be ingested back into the Monarch KG, potentially providing new, ‘non-obvious’ KG edges between phenotypes in different organisms. In the future, we will generate gene candidate predictions from Phenologs using a k-nearest neighbors approach described in Woods et al. (49).

In addition to enhancing Monarch's user-facing aspects, the next phase of Monarch will also focus on making our data and services more interoperable with existing software and data science standards. Monarch is always looking for ways to make our efforts more impactful, and our future plans are to design features that leverage disruptive technologies for this purpose. As highlighted above, we are actively developing ML technologies for applications in Monarch and wider use. The in-progress Monarch Assistant, which will combine the ability of LLMs to answer questions in plain language with Monarch's extensive KG and analysis algorithms, will open the door to an even broader community of basic, clinical, translational and lay-person users.

## Data Availability

The Monarch Initiative is deeply committed to Open Science, and we make all Monarch resources freely available to everyone, minimizing barriers to entry. We don’t require anyone to register to use our website, and we provide multiple avenues for access to and reuse of the data we curate and host, including APIs and bulk downloads. All of Monarch's code and standards are shared openly on GitHub, and the entirety of the Monarch platform is reproducible by others. The locations of our data resources are listed below. Monarch Data: data.monarchinitiative.org/monarch-kg-dev Zenodo Deposit: DOI: 10.5281/zenodo.8350685; zenodo.org/record/8 350 685 Monarch Mapping Commons: github.com/monarch-initiative/monarch-mapping-commons Mondo Mappings: github.com/monarch-initiative/mondo/tree/master/src/ontology/mappings Phenomics Integrated Ontology (PHENIO): github.com/monarch-initiative/phenio Semsimian: github.com/monarch-initiative/semsimian KGX Summarize Graph - kghub.org/kg-hub-dashboard/ (select Monarch KG) Monarch QC Reports: github.com/monarch-initiative/monarch-qc-reports Monarch KG QC Schema: monarch-initiative.github.io/monarch-kg-qc-schema/ Monarch Ingest Dashboard: github.com/monarch-initiative/monarch-qc Phenotypic Inference Evaluation Framework (PhEval): github.com/monarch-initiative/pheval Semantic Similarity Profile data model: incatools.github.io/ontology-access-kit/datamodels/similarity/index.html Licensing: The Monarch web application and its source code are licensed under the 3-Clause BSD License (opensource.org/licenses/BSD-3-Clause). Licensing information for the data sources and ontologies that make up the Monarch knowledge graph can be found on the sources page (monarch-initiative.github.io/monarch-ingest/Sources).
